# Multivariate Optimization of Ultrasound-Assisted Extraction of Phenolic Compounds from Apples

**DOI:** 10.3390/molecules31081314

**Published:** 2026-04-17

**Authors:** Francesca Melini, Sara Fasano, Valentina Melini

**Affiliations:** 1 CREA Research Centre for Food and Nutrition, Via Ardeatina 546, I-00178 Rome, Italy; francesca.melini@crea.gov.it; 2 Department for Innovation in Biological, Agro-Food and Forest Systems (DIBAF), University of Tuscia, Via C. de Lellis snc, I-01100 Viterbo, Italy; sara.fasano@studenti.unitus.it

**Keywords:** phenolic compounds, apples, ultrasound-assisted extraction, response surface methodology, phytochemicals

## Abstract

Apples (*Malus domestica* Borkh.) are among the most widely consumed fruits worldwide and represent a significant dietary source of phenolic compounds. Efficient extraction is a critical step for the isolation, characterization, and quantification of phenolic compounds. The extraction yield and composition are strongly influenced by multiple parameters, including solvent type and concentration, temperature, extraction time, solid-to-liquid ratio, and the presence and concentration of acidifying agents. This study aimed to optimize an ultrasound-assisted extraction (UAE) procedure using response surface methodology (RSM) to evaluate the effects of extraction temperature, solvent-to-sample ratio (SSR) and citric acid concentration on total phenolic content (TPC) and total flavonoid content (TFC). Statistical analysis showed that SSR and temperature were the most influential factors affecting phenolic recovery, while citric acid concentration exerted a secondary, interaction-driven effect. Optimization using a desirability function identified the operating conditions that maximized phenolic and flavonoid recovery: 55 °C, 10 mL/g SSR and 0.2% citric acid concentration. Model predictions were validated experimentally, confirming the reliability of the approach for TPC and TFC. Chlorogenic acid and flavan-3-ols, including monomers, such as catechin and epicatechin, and polymers such as procyanidins, were identified. Overall, the proposed approach provides a statistically supported framework for phenolic compound analysis in apples.

## 1. Introduction

Phenolic compounds constitute one of the most important classes of plant secondary metabolites, with more than 8000 structures identified to date. In plants, these compounds are synthesized primarily through the shikimate pathway, which leads to the formation of the phenylpropanoid (C6–C3) backbone, or through the acetate pathway, which contributes two-carbon building units for polymeric structures [1]. Their biosynthesis occurs during normal physiological development but can also be stimulated by environmental stressors such as mechanical injury, temperature fluctuations, ultraviolet radiation, or microbial attack. Structurally, phenolic compounds are characterized by at least one aromatic ring bearing one or more hydroxyl groups. This broad class encompasses a wide spectrum of molecules, ranging from low-molecular-weight phenolic acids to more structurally complex flavonoids [2].

Phenolic compounds are widely distributed in plant-derived foods. Fruits and vegetables constitute the primary dietary sources, although whole grains and pseudocereals also provide considerable amounts of these bioactive phytochemicals. The Phenol-Explorer database, a comprehensive resource compiling data on polyphenol concentrations in foods, includes information on approximately 500 individual phenolic compounds across more than 400 food items, corresponding to roughly 35,000 reported values. Moreover, scientific interest in food phytochemicals has grown substantially, as reflected by the increasing number of analytical studies published in recent years focusing on their identification and quantification. Phenolic content in plant foods is shaped by genetics, growing conditions, and processing, making robust methods essential for accurately evaluating their effects on food quality and bioactive compound content.

Apples (*Malus domestica* Borkh.) are among the most widely consumed fruits worldwide and represent a significant dietary source of phenolic compounds. The phenolic profile of apples includes hydroxycinnamic acids (mainly chlorogenic acid), flavan-3-ols (catechin and epicatechin), procyanidins, flavonols (quercetin glycosides), and dihydrochalcones (phloridzin). These compounds are unevenly distributed within the fruit, with higher concentrations typically found in the peel compared to the flesh. Efficient extraction is a critical step for the isolation, characterization, and quantification of phenolic compounds. The extraction yield and composition are strongly influenced by multiple parameters, including solvent type and concentration, temperature, extraction time, solid-to-liquid ratio, presence and concentration of acidifying agents, and particle size. Because phenolic compounds exhibit diverse polarity and stability profiles, the optimization of extraction conditions is necessary to maximize recovery while preventing degradation or structural modification. Although several studies have reported the extraction of phenolic compounds from apples [3,4,5,6], the systematic optimization of the process using a Box–Behnken design (BBD) and the evaluation of interactions among key extraction variables through Response Surface Methodology (RSM) remain underexplored.

RSM is a multivariate statistical and mathematical tool that enables the evaluation of the effects and interactions of independent variables on one or more response variables. Unlike traditional one-factor-at-a-time experiments, RSM provides a comprehensive understanding of process behavior with a reduced number of experimental trials. In phytochemical extraction research, RSM has been successfully applied to optimize operational parameters and to develop predictive second-order polynomial models. Such models allow not only the identification of optimal conditions but also the assessment of variable interactions and quadratic effects, which are particularly relevant in mass transfer-driven processes.

The aim of the present study was to identify more sustainable operational conditions for phenolic extraction from apples. To this aim, an ultrasound-assisted extraction procedure was optimized by evaluating the effects of selected independent variables—extraction temperature, citric acid concentration and solvent-to-liquid ratio—on total phenolic content and profile. The predicted optimal conditions were experimentally validated. The results of this study provide a systematic and statistically supported approach for maximizing phenolic recovery from apples, with potential applications in analytical standardization, quality evaluation of apple cultivars, and valorization of apple-derived products.

## 2. Results

### 2.1. Optimization of Phenolic Extraction by BBD

#### 2.1.1. Model Fitting and Statistical Validation for BBD

The experimental data obtained for the 15 runs of BBD are reported in Table 1. TPC ranged from 143.34 (run #1) to 200.03 (run #12) mg GAE/100 g fw; TFC ranged from 105.22 (run #4) to 144.94 (run #3) mg CE/100 g fw. Comparison of the data obtained in this study with those reported in the literature is not possible since, to the best of our knowledge, apples belonging to the ‘Renetta’ group have not been previously investigated for TPC and TFC. Moreover, the absence of official methods for phenolic compound extraction, together with the use of different extraction procedures, further hampers direct comparison of polyphenol contents across studies.

The experimental data (Table 1) were successfully fitted to a second-order response surface model to describe the effects of citric acid concentration (X_1_), extraction temperature (X_2_), and solvent-to-sample ratio (SSR, X_3_) on TPC and TFC.

For TPC, the quadratic model was highly significant (F = 32.20, *p* = 0.0007; Table 2), indicating that the model adequately describes the relationship between extraction variables and phenolic recovery. The coefficient of determination (R^2^ = 0.983, calculated from sums of squares) suggests that 98.3% of the variability in TPC was explained by the model, demonstrating excellent predictive capability. The adjusted model performance was further supported by the non-significant lack-of-fit test (*p* = 0.7586), confirming that the model error was primarily associated with random experimental variability rather than systematic bias.

Similarly, the TFC model was statistically significant (F = 66.82, *p* = 0.0001), indicating that the selected independent variables adequately describe the variation in flavonoid extraction within the investigated domain. The lack-of-fit test was not significant (*p* = 0.8465), confirming that the model provided an appropriate fit to the experimental data and that deviations were mainly attributable to random experimental error rather than model inadequacy.

The final empirical model describing TPC, expressed in terms of coded factors, is given by:

$$\mathrm{TPC}=+181.06+1.64X_{1}+6.64X_{2}+19.77X_{3}-0.24X_{1}X_{2}+7.07X_{1}X_{3}-2.30X_{2}X_{3}\;-4.49{X_{1}}^{2}-1.23{X_{2}}^{2}-5.64{X_{3}}^{2}$$

The coded model is particularly useful for evaluating the relative contribution of each factor to the response, as the magnitude of the regression coefficients reflects the strength of their influence. Larger absolute coefficient values indicate a greater impact on TPC variation. In this case, the coefficient associated with temperature (X_2_) and solvent-to-sample ratio (X_3_) appears numerically larger than that of citric acid concentration (X_1_), suggesting a stronger contribution of these variables to the predicted response. However, statistical significance must be interpreted in conjunction with the ANOVA results.

Furthermore, the inclusion of interaction (X_1_X_2_, X_1_X_3_, X_2_X_3_) and quadratic (X_1_^2^, X_2_^2^, X_3_^2^) terms allows the model to account for potential synergistic effects and curvature in the response surface, providing a more accurate representation of the extraction behavior within the investigated range.

The final empirical model describing TFC, expressed in terms of coded factors, is given by:

$$\mathrm{TFC}=+128.21-1.25X_{1}+10.54X_{2}+6.68X_{3}+5.80X_{1}X_{2}+7.42X_{1}X_{3}+1.77X_{2}X_{3}\;-3.56{X_{1}}^{2}-1.39{X_{2}}^{2}-0.45{X_{3}}^{2}$$

Similarly to TPC, the equation expressed in coded factors can be used to predict TFC values at specific levels of each independent variable within the investigated experimental domain.

Among the linear terms, temperature (X_2_) and solvent-to-sample ratio (X_3_) exhibit the largest positive coefficients, highlighting their strong influence in enhancing flavonoid extraction. In contrast, citric acid concentration (X_1_) shows a very small negative coefficient, confirming its limited and non-significant linear effect, as previously indicated by the ANOVA.

The interaction terms (X_1_X_2_, X_1_X_3_, and X_2_X_3_) display relatively modest positive coefficients, suggesting weak synergistic effects between variables. Likewise, the quadratic terms are all negative but small in magnitude, indicating only slight curvature in the response surface and the absence of pronounced optimal peaks within the studied range.

Overall, these findings suggest that TFC extraction is predominantly governed by the linear effects of temperature and solvent-to-sample ratio, while interaction and quadratic contributions remain secondary, resulting in a response that is largely linear across the investigated experimental conditions.

#### 2.1.2. Effect of BBD Independent Variables on TPC

Among the linear terms, temperature (X_2_) and solvent-to-sample ratio (X_3_) significantly affected TPC (*p* = 0.0037 and *p* < 0.0001, respectively), whereas citric acid concentration (X_1_) did not show a statistically significant linear contribution (*p* = 0.2613; Table 2).

The extremely high F-value associated with solvent-to-sample ratio (F = 232.19) indicates that this parameter is the dominant factor governing phenolic extraction efficiency. Increasing solvent volume enhances the concentration gradient between the plant matrix and extraction medium, thereby intensifying mass transfer. Under ultrasound-assisted conditions, improved solvent accessibility facilitates cavitation-induced cell wall disruption and accelerates solubilization of intracellular phenolics. Temperature also exerted a significant positive effect on TPC. The enhancement of phenolic recovery at higher temperatures can be attributed to reduced solvent viscosity, increased diffusivity, and intensified cavitation phenomena. Elevated temperatures improve bubble formation and collapse dynamics, promoting mechanical rupture of plant tissues and improved metabolite release. In contrast, citric acid concentration did not independently influence TPC in a linear manner within the investigated range. This suggests that moderate variations in acidification were insufficient to significantly alter phenolic solubility or stability when considered alone.

Among the interaction terms, only the interaction between citric acid concentration and solvent-to-sample ratio (X_1_X_3_) was statistically significant (*p* = 0.0120; Table 2). This indicates that the effect of solvent volume on TPC depends on the level of acidification. Mechanistically, this interaction suggests that solvent acidification may enhance phenolic stability and prevent oxidative degradation, particularly when sufficient solvent is available to efficiently extract and stabilize the released compounds. The synergistic X_1_X_3_ effect highlights the importance of simultaneously optimizing solvent composition and solvent quantity. The X_1_X_2_ and X_2_X_3_ interactions were not significant (*p* = 0.9028 and *p* = 0.2646, respectively; Table 2), indicating that temperature operates largely independently from acid concentration and solvent volume within the studied region.

Regarding curvature, the quadratic term associated with solvent-to-sample ratio (X_3_^2^) was significant (*p* = 0.0317; Table 2), demonstrating the existence of a non-linear relationship between solvent volume and phenolic extraction. This finding indicates that TPC does not increase indefinitely with solvent addition but rather reaches an optimum within the experimental domain. Excessively high solvent volumes may reduce cavitation intensity per unit volume or dilute extracted phenolics, thereby limiting further increases in measured TPC. The quadratic term for citric acid concentration (X_1_^2^) showed a borderline effect (*p* = 0.0657; Table 2), suggesting a tendency toward curvature that may become significant in a broader experimental range. Conversely, temperature did not exhibit significant quadratic behavior (*p* = 0.5492; Table 2), indicating a predominantly linear contribution within the investigated conditions.

Taken together, the statistical results indicate that solvent-to-sample ratio is the primary driving factor controlling phenolic extraction under ultrasound-assisted conditions, followed by temperature. Citric acid concentration plays a modulatory role, mainly through its interaction with solvent volume rather than through an independent linear effect.

The combination of significant linear and quadratic effects for solvent-to-sample ratio suggests that phenolic recovery is governed by a balance between enhanced mass transfer and system dilution effects. The model therefore captures both mechanistic aspects of ultrasound-assisted extraction: cavitation-enhanced matrix disruption, and diffusion-driven solute transfer governed by solvent availability.

Overall, the validated quadratic model provides a statistically robust and mechanistically coherent description of TPC extraction behavior, enabling reliable prediction and optimization of phenolic recovery from the apple matrix.

**Figure 1:**
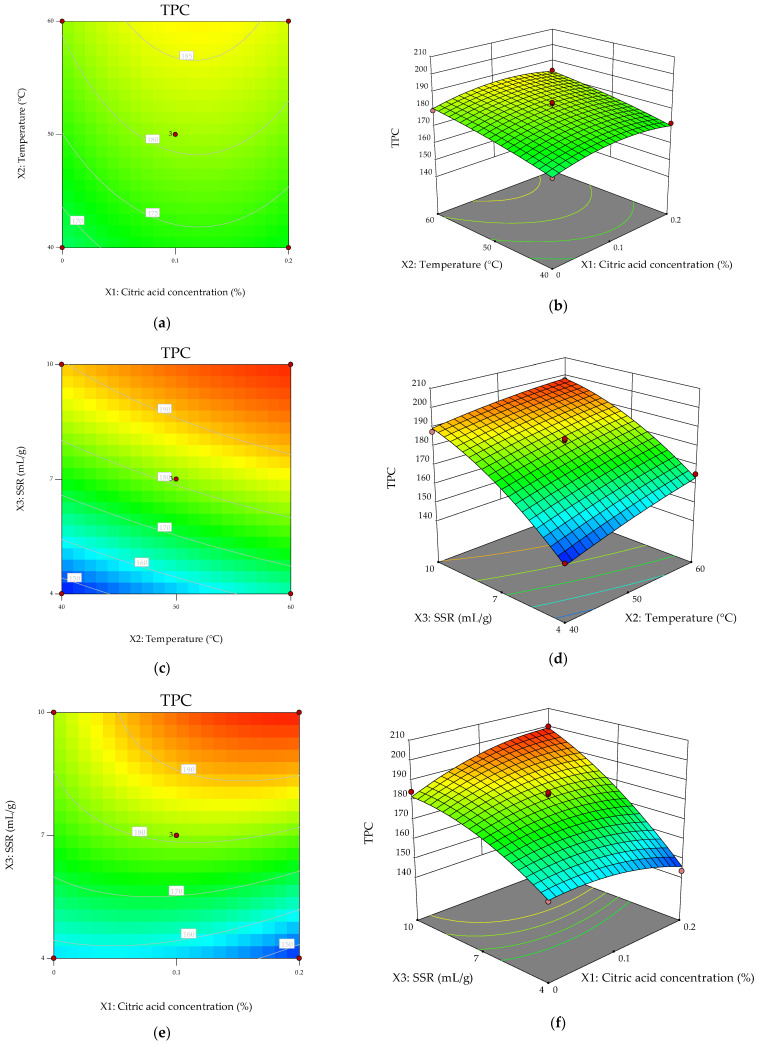
Response surface and contour plots showing the modeled effects of process variables on total phenolic extraction yield. (**a**,**b**): contour plot and response surface relative to the interaction X_1_X_2_; (**c**,**d**): contour plot and response surface relative to the interaction X_2_X_3_; (**e**,**f**): contour plot and response surface relative to the interaction X_1_X_3_.

To better elucidate the combined effects of the extraction variables on TPC, contour plots and 3-D plots were generated based on the fitted quadratic model. The three-dimensional response surfaces and corresponding contour plots (Figure 1) provided visual confirmation of the statistical trends highlighted by ANOVA and allowed deeper interpretation of the extraction dynamics under ultrasound-assisted conditions.

The response surface describing the combined effect of temperature (X_2_) and solvent-to-sample ratio (X_3_) (Figure 1d) showed a pronounced inclination along the X_3_-axis, confirming the dominant linear effect of solvent volume (F = 232.19, *p* < 0.0001; Table 2). The contour lines appeared predominantly elliptical with slight curvature along the X_3_ direction, consistent with the significant quadratic term (X_3_^2^, *p* = 0.0317). This topology indicates the presence of an optimal solvent-to-sample ratio, beyond which further solvent addition does not proportionally enhance TPC.

From a mechanistic standpoint, increasing solvent volume initially intensifies the concentration gradient between the plant matrix and extraction medium, promoting diffusion-driven mass transfer. However, excessive solvent volume may attenuate cavitation intensity per unit volume and dilute extracted phenolics, thereby explaining the observed curvature.

The temperature–SSR surface further illustrates that temperature exerts a positive but secondary effect relative to solvent ratio. The surface rises with increasing temperature, consistent with the significant linear contribution of X_2_ (*p* = 0.0037), yet lacks strong curvature along the temperature axis, in agreement with the non-significant X_3_^2^ term. This suggests that within the investigated range, temperature enhancement primarily improves extraction through increased diffusivity and improved cavitation dynamics rather than through threshold-dependent mechanisms.

The contour plot corresponding to citric acid concentration (X_1_) and solvent-to-sample ratio (X_3_) (Figure 1e) displayed elliptical contours tilted along the diagonal axis, reflecting the significant X_1_X_3_ interaction (*p* = 0.0120). This confirms that the influence of solvent volume on TPC is modulated by acid concentration. At higher solvent ratios, moderate acidification appears to enhance phenolic recovery more effectively than at lower solvent volumes. Such behavior may be attributed to improved stabilization of phenolic compounds under mildly acidic conditions, particularly when sufficient solvent is available to prevent localized saturation or oxidative degradation.

Conversely, the response surface for the X_1_–X_2_ combination exhibited nearly parallel contour lines (Figure 1b), supporting the non-significant X_1_X_2_ interaction. This indicates that temperature operates largely independently of acid concentration in determining TPC within the studied domain.

Overall, the graphical analysis corroborates the statistical findings and highlights solvent-to-sample ratio as the primary determinant of phenolic recovery, with temperature acting as a complementary enhancing factor and citric acid concentration exerting a conditional, interaction-driven influence. The combined statistical and graphical interpretation demonstrates that ultrasound-assisted phenolic extraction from the apple matrix is governed by the interplay between cavitation-enhanced structural disruption and diffusion-controlled solute transfer, optimized within a defined solvent–thermal window.

#### 2.1.3. Effect of BBD Independent Variables on TFC

For TFC, temperature (*p* < 0.0001) and SSR (*p* < 0.0001) were identified as highly significant linear variables, whereas citric acid concentration (X_1_) did not show a statistically significant effect at the 95% confidence level (*p* = 0.0888), although a slight trend was observed (Table 2).

These results indicate that flavonoid extraction was primarily governed by thermal effects and solvent availability. The strong dependence on temperature can be attributed to enhanced solubility and diffusion of flavonoids, as well as improved cell disruption under combined thermal and ultrasonic cavitation effects. Increased temperature may also intensify ultrasound cavitation effects, promoting cell wall disruption and facilitating metabolite release. The significant effect of the solvent-to-sample ratio indicates that solvent availability plays an important role in flavonoid extraction, likely by enhancing mass transfer and maintaining a favorable concentration gradient between the plant matrix and the extraction medium. The higher F-values associated with temperature and solvent-to-sample ratio further confirm their dominant contribution to TFC extraction efficiency. In contrast, citric acid concentration did not significantly influence TFC, suggesting that flavonoids were relatively insensitive to moderate acidification within the studied range.

Significant interaction terms were observed for X_1_X_2_ (*p* = 0.0009) and X_1_X_3_ (*p* = 0.0003), indicating that the effect of citric acid concentration depends on both temperature and solvent-to-sample ratio (Table 2). This suggests the presence of synergistic mechanisms, where acidification may enhance flavonoid extraction under specific thermal and solvent conditions. The X_2_X_3_ interaction was not statistically significant (*p* = 0.0872), indicating that temperature and solvent ratio mainly act independently of each other (Table 2).

Regarding curvature effects, the quadratic term X_1_^2^ was statistically significant (*p* = 0.0094), indicating a non-linear relationship between citric acid concentration and TFC (Table 2). This suggests the existence of an optimal acid concentration within the studied range. In contrast, X_2_^2^ and X_3_^2^ were not significant (*p* > 0.05), indicating that temperature and solvent-to-sample ratio exhibited predominantly linear effects within the investigated experimental domain.

Overall, the RSM model adequately described flavonoid extraction behavior and identified temperature and solvent availability as key determinants of TFC recovery, while also highlighting the contribution of interaction effects and partial curvature associated with citric acid concentration.

**Figure 2:**
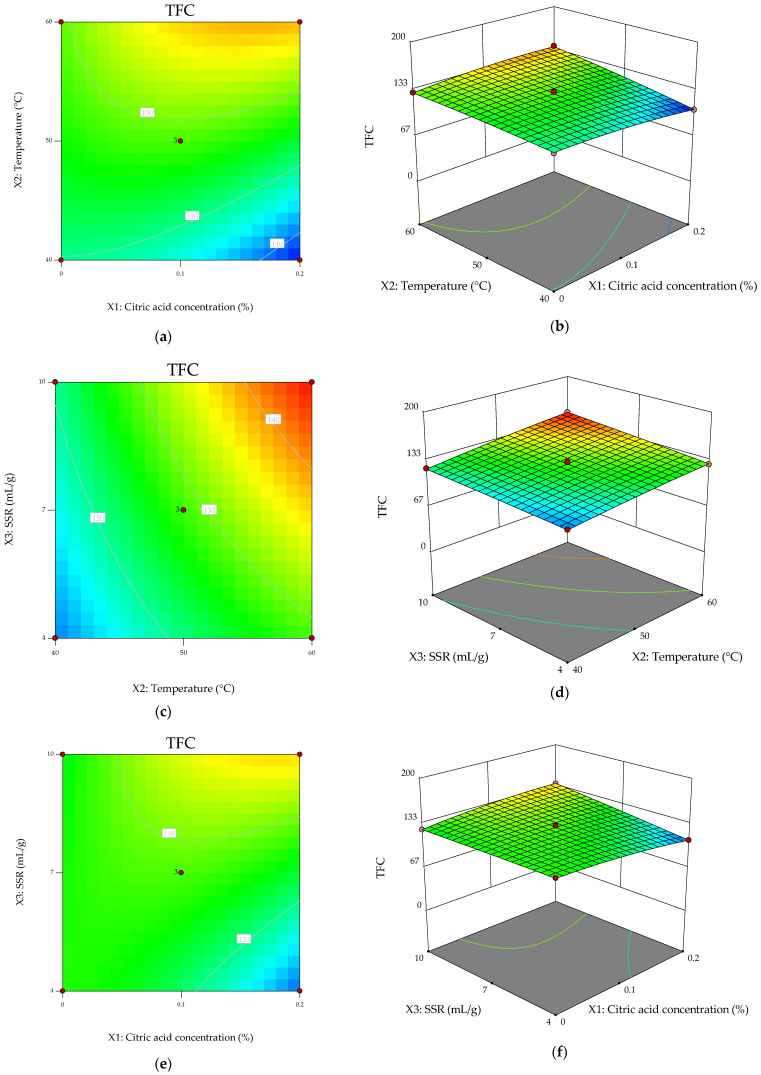
Response surface and contour plots showing the modeled effects of process variables on total flavonoid yield. (**a**,**b**): contour plot and response surface relative to the interaction X_1_X_2_; (**c**,**d**): contour plot and response surface relative to the interaction X_2_X_3_; (**e**,**f**): contour plot and response surface relative to the interaction X_1_X_3_.

The three-dimensional response surfaces and corresponding contour plots (Figure 2) visually corroborate the statistical outcomes of the ANOVA model and confirm the combined linear and interaction-driven behavior of TFC within the investigated experimental domain.

The response surface describing the combined effect of citric acid concentration and temperature (Figure 2b) shows a clear gradient along the temperature axis, accompanied by noticeable curvature along the citric acid axis. The contour lines deviate from parallelism and exhibit slight bending, confirming a significant interaction between these variables (X_1_X_2_; *p* = 0.0009), as well as a significant quadratic effect of citric acid concentration (X_1_^2^; *p* = 0.0094) (Figure 2a). These results indicate that the influence of citric acid on flavonoid extraction is dependent on temperature, suggesting that acidification enhances flavonoid stability or solubility more effectively at elevated temperatures.

The response surface describing the interaction between temperature and solvent-to-sample ratio displays a predominantly inclined plane with slightly curved contour lines (Figure 2d). This topology reflects the strong positive linear effects of temperature (*p* < 0.0001) and solvent-to-sample ratio (*p* < 0.0001), along with a weak but non-significant interaction (X_2_X_3_, *p* = 0.0872). The steady upward slope along both axes indicates that increasing either parameter enhances flavonoid recovery. Mechanistically, this behavior suggests that TFC extraction is primarily governed by diffusion-controlled mass transfer, facilitated by increased solute solubility at higher temperatures and improved solvent penetration, alongside cavitation-assisted matrix disruption.

The response surface for citric acid concentration and solvent-to-sample ratio (Figure 2f) further highlights the dominant role of solvent volume, while also revealing interaction effects. A pronounced slope is observed along the SSR axis, combined with curvature and slight contour distortion along the citric acid direction. This behavior is consistent with the significant interaction term (X_1_X_3_; *p* = 0.0003), indicating that the effect of acidification becomes more pronounced at higher solvent volumes. This suggests a synergistic contribution of solvent availability and acid conditions in enhancing flavonoid extraction efficiency.

Overall, the TFC response surfaces exhibit moderate curvature and significant interaction patterns, particularly involving citric acid concentration. Although temperature and solvent-to-sample ratio remain the primary driving factors, the presence of significant interactions and quadratic effects indicates a more complex extraction mechanism than purely additive behavior.

This behavior suggests that flavonoid extraction is governed by both linear and synergistic effects, likely arising from the interplay between solubility enhancement, matrix disruption, and compound stability under varying extraction conditions.

#### 2.1.4. Multi-Response Optimization Using the Desirability Function and Experimental Validation of the Optimized Conditions

To validate the optimization results obtained through the desirability function approach (Desirability = 1.00), experiments were performed at the predicted optimal levels of the independent variables: X_1_ = 0.2%, X_2_ = 55 °C, X_3_ = 10 mL/g). The numerical optimization predicted that, under these optimal conditions, TPC would reach 200.99 mg GAE/100 g fw, while TFC was estimated at 145.04 mg CE/100 g fw. The predicted values indicate that the selected operating conditions simultaneously maximize the recovery of phenolic and flavonoid compounds within the studied experimental domain.

To confirm model adequacy, experimental runs were conducted under the optimized conditions, and the obtained results were compared with the predicted values. Experimental validation yielded a TPC value of 226.29 mg GAE/100 g fw and a TFC value of 130.08 mg CE/100 g fw.

For TFC, the experimentally obtained value fell within the 95% prediction interval (132.97–157.11 mg CE/100 g fw), confirming the adequacy and predictive reliability of the regression model for flavonoid extraction. The relatively small deviation from the predicted mean further supports the robustness of the model within the optimized domain.

Similarly, the experimental value obtained for TPC fell within the 95% prediction interval (174.50–227.53 mg GAE/100 g fw), indicating that the model adequately predicts phenolic recovery when experimental variability is taken into account. Although the observed value is close to the upper bound of the interval, the behavior suggests that the predicted optimum lies near a region of maximum response, where small variations in process conditions may lead to noticeable changes in extraction yield. In ultrasound-assisted extraction systems, such nonlinear behavior is frequently associated with time-dependent cavitation dynamics and diffusion-controlled mass transfer mechanisms. Since extraction time was kept constant during BBD, the possibility remained that further enhancement—or stabilization—of phenolic recovery could be achieved through kinetic optimization.

### 2.2. Phenolic Compounds Identified in Extract at Optimal Conditions

**Figure 3:**
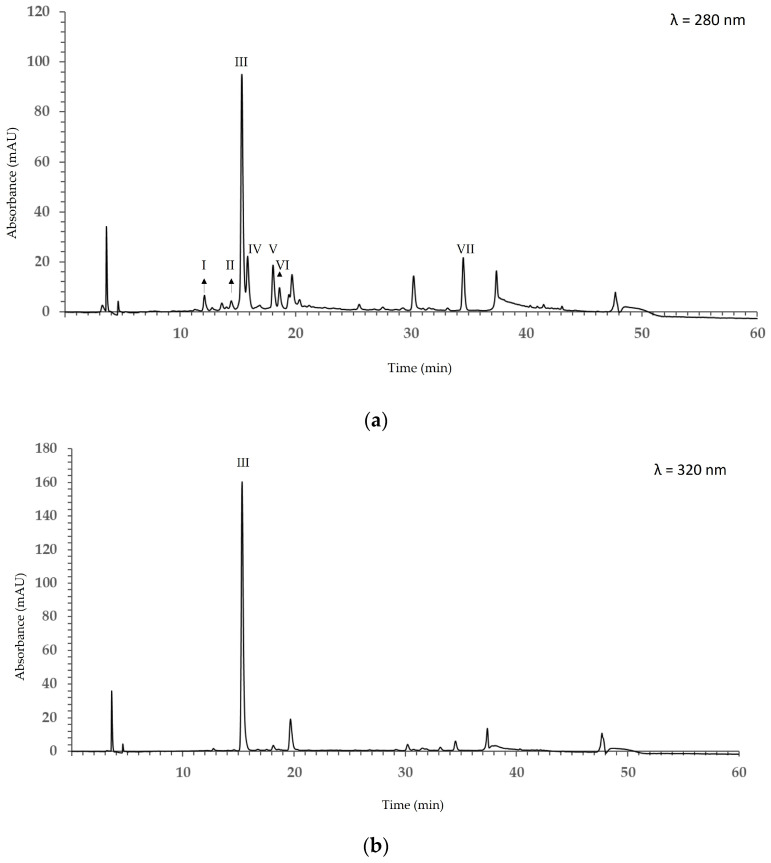
Chromatograms of apple extract obtained under optimal extraction conditions recorded at 280 nm (**a**) and 320 nm (**b**). Peaks are identified as follows: I: procyanidin B1, II: (+)-catechin, III: chlorogenic acid, IV: procyanidin B2, V: (−)-epicatechin, VI: procyanidin C1, VII: phloridzin.

In the extract obtained under optimal extraction conditions, a total of seven phenolic compounds (I–VII) were identified by RP-HPLC (Figure 3) at 280 nm and 320 nm.

In detail, procyanidin B1, procyanidin B2, and procyanidin C1 were detected at 12.0, 15.8, and 18.6 min, respectively. The flavan-3-ols (+)-catechin and (−)-epicatechin were identified at 14.2 and 18.0 min, respectively. Chlorogenic acid was detected at 15.2 min, while the dihydrochalcone phloridzin (phloretin 2′-O-glucoside) was detected at 34.23 min. In the chromatogram recorded at 320 nm, only chlorogenic acid was observed. Following peak-area normalization, the percentage composition of the mixture was calculated: procyanidin B1, 2.5%; catechin, 1.9%; chlorogenic acid, 63.8%; procyanidin B2, 9.9%; epicatechin, 8.1%; procyanidin C1, 4.0%; and phloridzin, 9.7%.

## 3. Discussion

The present study provides a systematic and statistically robust optimization of phenolic compound extraction from apples through the integration of RSM with UAE. Unlike conventional one-factor-at-a-time approaches, which neglect interaction and curvature effects, RSM enabled the simultaneous evaluation of linear, quadratic, and interaction terms, thereby offering a more comprehensive understanding of extraction dynamics. To date, only Alberti et al. [7] have applied RSM to apple phenolic extraction; however, their work was limited to conventional solid–liquid extraction and did not incorporate process intensification strategies such as ultrasound. Therefore, the present study contributes to the existing literature by combining UAE with multivariate statistical optimization and by providing a detailed interpretation of factor interactions within the investigated experimental domain, rather than introducing a fundamentally new extraction technique.

The statistical analysis clearly identified SSR as the dominant factor governing TPC, with temperature exerting a secondary but significant effect. The strong influence of SSR highlights the importance of maintaining an adequate concentration gradient to promote diffusion-controlled mass transfer, particularly under ultrasound conditions where cavitation enhances cell wall disruption and intracellular release. While ultrasound-assisted extraction is known to promote matrix disruption through cavitation phenomena, the present study did not include a direct comparison with non-ultrasound extraction; therefore, this effect is inferred based on established literature. The significant quadratic contribution of SSR further indicates the existence of an optimal solvent window, beyond which dilution effects or reduced cavitation efficiency may limit additional gains in extraction yield. In contrast, TFC exhibited predominantly linear behavior, suggesting that this subclass of phenolics responds more uniformly to thermal and solvent variations within the investigated range. The weaker curvature and interaction effects observed for TFC may reflect the comparatively greater structural stability of flavonoids under moderately acidic and thermal conditions.

An additional innovative aspect of this work lies in the evaluation of solvent acidification using citric acid. With the exception of isolated investigations on apple peel [8], the influence of solvent acidification on phenolic extraction from whole apple matrices has received limited attention. In the present study, citric acid concentration did not exert a strong independent linear effect but showed a significant interaction with SSR for TPC. This suggests that acidification plays a stabilizing rather than solubilizing role, particularly under conditions of sufficient solvent availability. Mild acidification may contribute to the suppression of oxidative degradation and stabilization of phenolic structures during extraction, especially in matrices prone to enzymatic browning. Moreover, citric acid is a biodegradable, food-grade, and environmentally benign organic acid compared to mineral acids traditionally used in phytochemical extraction. Although methanol was employed as the primary extraction solvent due to its well-established efficiency in analytical applications, the use of a mild organic acid contributes to improving the overall safety and compatibility of the extraction system.

Importantly, the optimized protocol demonstrated that efficient phenolic recovery can be achieved using substantially smaller sample masses than those typically reported in the literature. Previous studies have commonly employed 5–20 g of fresh material [9,10,11,12], requiring correspondingly large solvent volumes. Such approaches increase solvent consumption, operational cost, and environmental impact, and may complicate downstream concentration and analytical steps. The ability to obtain high recovery from only 0.5 g of fresh sample under optimized UAE conditions represents a meaningful advancement in methodological efficiency. Analytical methods requiring small sample quantities have several advantages, including minimizing solvent and reagent use, enhancing laboratory safety and allowing multiple analyses or replicates from the same sample, thus making the procedure more sustainable, practical, and efficient.

The choice of cryogenic sample preparation further strengthens the methodological framework and supports the sustainability framework. Liquid nitrogen treatment ensures rapid enzymatic inactivation and metabolic arrest, thereby preserving the native phenolic profile prior to extraction. Although freeze-drying is widely used for plant matrices, apples are infrequently subjected to lyophilization before phenolic extraction due to their high moisture and sugar content, which often necessitates extended or repeated drying cycles to achieve structural stability. Such additional processing increases energy demand and may alter matrix microstructure, potentially influencing solvent penetration and mass transfer kinetics. Cryogenic stabilization therefore represents a more direct and less artifact-prone approach for preserving labile phenolic constituents while minimizing pre-analytical modification of the matrix.

Overall, the combined statistical and mechanistic interpretation indicates that ultrasound-assisted phenolic extraction from apples is governed by the balance between cavitation-enhanced structural disruption and diffusion-driven solute transfer, optimized within a defined solvent–thermal domain. Within the limits of the experimental design, the validated RSM model provides predictive capability and practical guidance for maximizing phenolic recovery under controlled laboratory conditions, while reducing solvent usage and processing time. Beyond its analytical relevance, the optimized framework may support future applications in food quality assessment, functional ingredient development, and valorization of apple-based matrices.

HPLC analysis enabled the identification of specific phenolic compounds in the apple extract. Chlorogenic acid was the most abundant, consistent with literature data [13]. Hydroxycinnamic acids, mainly represented by chlorogenic acid, commonly account for 70–80% of phenolic compounds in *Malus domestica* [13]. Flavan-3-ols, including catechin, epicatechin, and procyanidins, accounted for approximately 25% of the identified phenolics. (−)-Epicatechin was confirmed as the predominant flavan-3-ol (8.1%), typically occurring at concentrations two- to four-fold higher than (+)-catechin in both peel and pulp; a similar ratio was also observed under the extraction conditions applied in this study. The slightly acidic nature of the extraction solvent may have enhanced the recovery of catechins. In general, (+)-catechin and (−)-epicatechin are more efficiently extracted in mildly acidic hydroalcoholic solvents. Acidic conditions suppress polyphenol oxidase and peroxidase activity, which would otherwise oxidize catechins into brown polymeric products, while simultaneously protecting the catechol moieties from oxidative degradation [14,15]. In contrast, the percentage of phloridzin in this study was about twofold higher than values reported in recent studies in the literature. This discrepancy may be due to variations in apple cultivar, fruit tissue analyzed (peel vs. flesh), and the extraction methodology employed.

## 4. Materials and Methods

### 4.1. Chemicals and Reagents

Folin–Ciocalteu reagent, calcium chloride, citric acid, aluminum chloride, sodium nitrate, sodium hydroxide, and RPE-grade methanol were obtained from Carlo Erba Reagents (Milan, Italy). Analytical standards, including (+)-catechin, chlorogenic acid, (−)-epicatechin, gallic acid, phloridzin, procyanidin B1, procyanidin B2, and procyanidin C, were supplied by Extrasynthèse (Genay, France) and Sigma-Aldrich (St. Louis, MO, USA).

High-performance liquid chromatography (HPLC) analyses were performed using HPLC-grade solvents, and ultrapure water was produced with a Milli-Q purification system (Millipore Corporation, Burlington, MA, USA).

### 4.2. Sample Preparation

Apple samples were prepared as follows. ‘Renetta’ cv apples were purchased from different supermarkets in Rome (Italy) in October 2025. The ‘Renetta’ cultivar was selected due to its relatively high phenolic content compared to many commercial dessert cultivars, and well-characterized phytochemical profile, including hydroxycinnamic acids, flavan-3-ols, and dihydrochalcones. This makes the ‘Renetta’ cv particularly suitable for extraction optimization studies, as higher baseline levels allow clearer evaluation of extraction efficiency and variable effects. Moreover, its susceptibility to enzymatic browning makes it a suitable model matrix for evaluating extraction efficiency and the effectiveness of solvent acidification in limiting oxidative degradation. The widespread commercial availability of this cultivar further supports the reproducibility and practical relevance of the study.

The ‘Renetta’ apples were washed and wiped with a paper towel, and the core was removed using an apple slicer. They were then chopped under liquid nitrogen and finely ground using an IKA A 11 basic analytical mill (IKA-Werke GmbH, Staufen, Germany), which was also pre-treated with liquid nitrogen to prevent sample heating and degradation. The resulting powder was stored at −80 °C until further analysis.

### 4.3. Experimental Design

#### 4.3.1. Variable Selection

The experimental design of this study was developed using the Design Of Experiments (DOE) software package, Design Expert (version 10, Stat-Ease Inc., Minneapolis, MN, USA). Response Surface Methodology (RSM) was applied to determine the optimal levels of each independent variable and their combined effects to maximize the recovery of phenolic compounds. Total phenolic content (TPC) and total flavonoid content (TFC) were selected as responses (dependent variables).

Three independent variables were selected: citric acid concentration (X_1_, % *w*/*v*), extraction temperature (X_2_, °C), and solvent-to-sample ratio (X_3_, mL/g). Each factor (independent variable) was evaluated at three coded levels, corresponding to −1 (low level), 0 (central level), and +1 (high level). The actual and coded values of the variables are presented in Table 3. Sample weight and extraction time were kept constant at 0.5 g *fw* and 30 min, respectively, for all runs.

#### 4.3.2. Box–Behnken Designs and Regression Equation

A second-order polynomial model was applied to describe the relationship between the experimental variables and the measured responses:

(1) $$Y=\;\beta_{0}+\sum_{i=1}^{3}\beta_{i}\;X_{i}+\sum_{i=1}^{3}\beta_{\mathrm{ii}}\;X_{i}^{2}+\;\sum_{i=1}^{3}\;\sum_{j=1}^{3}\beta_{\mathrm{ij}}\;X_{i}\;X_{j}\;$$

In this equation, *Y* represents the response variable (e.g., TPC and TFC). The coefficients β_n_ are the regression parameters: β_0_ is the intercept, β_i_ represents the linear effects, β_ii_ accounts for quadratic effects, and β_i__j_ reflects interaction effects between variables. *X_i_* and *X_j_* denote the independent variables. The term *k* corresponds to the number of independent variables evaluated (k = 3).

Regression analysis and analysis of variance (ANOVA) were conducted to determine the significance of the model and its coefficients. Statistical significance was evaluated at a confidence level of 95% (*p* < 0.05). The goodness of fit of the polynomial equation was evaluated using the coefficient of determination (R^2^), where values approaching 1 indicate strong predictive capability. Additionally, the adjusted R^2^ (Adj. R^2^) was calculated to further examine model reliability.

Within the regression analysis, both the F-value and the lack-of-fit (LOF) test were evaluated at a significance level of *p* = 0.05. The predicted responses generated by the regression models were illustrated through contour plots and three-dimensional surface graphs.

For process optimization, the composite desirability function implemented in the Stat-Ease Optimizer was applied. The three independent variables (X_1_, X_2_, and X_3_) were simultaneously optimized with the objective of maximizing both response variables (TPC and TFC). The adequacy of the predictive model was subsequently verified by conducting additional extraction experiments under the identified optimal conditions.

### 4.4. Ultrasound-Assisted Extraction of Free Phenolic Compounds

Free phenolic compounds were extracted using an ultrasound-assisted procedure, as outlined hereafter. A defined amount of fresh sample was placed into a PYREX™ screw-cap culture tube, and a known volume of extraction solvent was added, according to the BBD. The extraction solvent consisted of methanol:water (80:20 *v*/*v*), either non-acidified or supplemented with citric acid at concentrations established by the experimental design (Table 3).

The tubes were then placed in an ultrasonic bath (Elmasonic S 100 H, Elma Schmidbauer GmbH, Singen, Germany) operating at 37 kHz. Prior to sonication, the samples were allowed to equilibrate in the water bath for 2 min to reach the set temperature. Throughout the extraction process, the bath temperature was monitored using a laboratory glass thermometer to ensure stable thermal conditions [8,16,17]. Extractions were performed individually and in randomized order in accordance with the experimental design.

After sonication, the extracts were refrigerated at +4 °C for 10 min and then centrifuged using a mini centrifuge LLG uniCFUGE 5 (LLG Labware, Teltow, Germany). The resulting supernatants were carefully collected and stored at −80 °C until spectrophotometric (TPC and TFC assays) and chromatographic (HPLC) analysis.

### 4.5. Determination of Total Phenolic Content and Total Flavonoid Content

Total phenolic content was quantified using the Folin–Ciocalteu colorimetric assay according to the method reported by Sompong et al. [18] and scaled down by Melini et al. [16,17]. In brief, a measured volume of the extract was mixed with Folin–Ciocalteu reagent previously diluted with water (1:10, *v*/*v*), and the reaction pH was adjusted by adding sodium carbonate solution (75 g/L). The tubes were then incubated in a water bath at 50 °C for 10 min. After cooling to room temperature, absorbance was recorded at 760 nm using a reagent blank as reference. The results were expressed as milligrams of gallic acid equivalents per 100 g of sample on a fresh weight basis (mg GAE/100 g fw).

Total flavonoid content (TFC) was determined using a colorimetric assay based on the method described by Alshikh et al. [19], with slight modifications outlined by Melini et al. [20]. Briefly, an aliquot of the previously prepared phenolic extract was neutralized and mixed with 5% (*w*/*v*) sodium nitrite solution. After 5 min of reaction, 10% (*w*/*v*) aluminum chloride solution was added. The reaction was finalized by adding 1 M sodium hydroxide and distilled water, followed by thorough mixing. The mixtures were kept at room temperature in the dark for 15 min, after which absorbance was measured at 510 nm against a reagent blank. All analyses were carried out in triplicate (*n* = 3). Quantification was performed using a catechin standard curve, and results were expressed as milligrams of catechin equivalents per 100 g of sample on a fresh weight basis (mg CE/100 g fw).

### 4.6. HPLC Analysis of the Extract Obtained Under Optimal Extraction Conditions

Phenolic compounds and flavonoids in the extract obtained at optimal conditions were analyzed using a Varian ProStar HPLC system (Varian Inc., Walnut Creek, CA, USA) equipped with a UV–VIS detector. Chromatographic separation was achieved on an Inertsil^®^ ODS-3 reversed-phase column (250 × 4.6 mm, 5 μm particle size; GL Sciences Inc., Tokyo, Japan).

The mobile phase consisted of water containing 2.5% acetic acid (solvent A) and acetonitrile (solvent B), delivered in gradient mode. The total run time was 60 min, with the following gradient program: 3% B at 0 min; 9% B at 5 min; 16% B at 15 min; 25% B at 33 min; 100% B from 35 to 43 min; and re-equilibration to 3% B at 45 min.

The flow rate was maintained at 1 mL/min up to 33 min, increased to 1.2 mL/min until 35 min, and then returned to 1 mL/min; the column temperature was set at 40 °C. The temporary increase in flow rate was applied to achieve better peak shape in the chromatogram. Prior to injection, all samples were filtered through 0.22 μm membrane filters. Based on the UV–VIS spectral characteristics of the reference standards, chromatograms were monitored at 280 and 320 nm.

The percentage composition of the mixture was determined by peak area normalization.

### 4.7. Statistical Analysis

All statistical analyses were carried out using Minitab^®^ Pro 18 (Minitab Inc., State College, PA, USA). Experimental data were additionally processed using Microsoft^®^ Excel^®^ for Windows 365 (version 2103). Response surface plots, contour diagrams, and three-dimensional graphs were generated with Design-Expert^®^ software (version 10, Stat-Ease, Inc., Minneapolis, MN, USA).

## 5. Conclusions

This study presents an efficient and environmentally sustainable framework for optimizing phenolic compound extraction from fresh apples through the integration of response surface methodology and ultrasound-assisted extraction. The results identified solvent-to-sample ratio and temperature as the primary determinants of phenolic recovery.

The developed method enables high phenolic recovery using reduced sample mass and lower solvent consumption, while the use of citric acid and cryogenic sample preparation helped preserve phenolic stability and minimize pre-analytical degradation. Overall, the proposed approach provides a sustainable, reproducible, and scalable strategy for phenolic extraction with potential applications in food quality analysis and functional product development.

## Figures and Tables

**Table 1:** Experimental design and results of BBD-RSM.

Std Order	Run Order	Citric Acid Concentration (%)	Temperature (°C)	SSR (mL/g)	TPC (mg GAE/100 g fw)	TFC (mg CE/100 g fw)
6	1	0.2	50	4	143.34 ± 2.19	108.86 ± 7.93
4	2	0.2	60	7	183.85 ± 1.87	139.18 ± 17.69
12	3	0.1	60	10	197.22 ± 1.89	144.94 ± 5.06
2	4	0.2	40	7	171.91 ± 0.44	105.22 ± 12.41
9	5	0.1	40	4	146.55 ± 1.42	111.32 ± 3.79
15	6	0.1	50	7	175.84 ± 3.77	126.61 ± 1.28
14	7	0.1	50	7	184.07 ± 4.78	130.63 ± 1.28
13	8	0.1	50	7	183.27 ± 4.89	127.97 ± 1.17
10	9	0.1	60	4	165.32 ± 1.61	127.58 ± 2.14
7	10	0	50	10	184.39 ± 0.93	124.67 ± 0.53
11	11	0.1	40	10	187.67 ± 1.82	121.60 ± 3.15
8	12	0.2	50	10	200.03 ± 0.52	136.63 ± 7.93
3	13	0	60	7	179.26 ± 8.81	129.67 ± 3.12
1	14	0	40	7	166.37 ± 0.40	118.92 ± 0.11
5	15	0	50	4	155.97 ± 4.05	126.25 ± 0.62

SSR: Solvent-to-Sample Ratio; TPC: Total Phenolic Content; TFC: Total Flavonoid Content.

**Table 2:** Analysis of variance (ANOVA) of the models for TPC and TFC in BBD.

	Source	SS	df	MS	F Value	*p*-Value	
TPC Model							
	Model	3901.12	9	433.46	32.20	0.0007	significant
	X_1_-Citric acid concentration	21.58	1	21.58	1.60	0.2613	
	X_2_-Temperature	353.10	1	353.10	26.23	0.0037	
	X_3_-SSR	3125.71	1	3125.71	232.19	<0.0001	
	X_1_X_2_	0.22	1	0.22	0.02	0.9028	
	X_1_X_3_	199.84	1	199.84	14.85	0.0120	
	X_2_X_3_	21.23	1	21.23	1.58	0.2646	
	X_1_^2^	74.27	1	74.27	5.52	0.0657	
	X_2_^2^	5.55	1	5.55	0.41	0.5492	
	X_3_^2^	117.54	1	117.54	8.73	0.0317	
	Residual	67.31	5	13.46			
	Lack of Fit	26.10	3	8.70	0.42	0.7586	not significant
TFC Model							
	Model	1677.56	9	186.40	66.82	0.0001	significant
	X_1_-Citric acid concentration	12.40	1	12.40	4.45	0.0888	
	X_2_-Temperature	888.40	1	888.40	318.46	<0.0001	
	X_3_-SSR	357.35	1	357.35	128.10	<0.0001	
	X_1_X_2_	134.62	1	134.62	48.26	0.0009	
	X_1_X_3_	220.61	1	220.61	79.08	0.0003	
	X_2_X_3_	12.57	1	12.57	4.51	0.0872	
	X_1_^2^	46.90	1	46.90	16.81	0.0094	
	X_2_^2^	7.18	1	7.18	2.58	0.1694	
	X_3_^2^	0.75	1	0.75	0.27	0.6273	
	Residual	13.95	5	2.79			
	Lack of Fit	4.00	3	1.33	0.27	0.8465	not significant

SS: sum of squares; df: degree of freedom; MS: mean square; F: variance; *p*: test statistics.

**Table 3:** Range and levels of experimental variables of the experiments.

	Factor	Symbol	Levels		
			Low (−1)	Intermediate (0)	High (+1)
BBD	Citric acid concentration (%)	X_1_	0	0.1	0.2
	Temperature (°C)	X_2_	40	50	60
	SSR (mL/g)	X_3_	4	7	10

SSR: solvent-to-sample ratio.

## Data Availability

The original contributions presented in this study are included in the article. Further inquiries can be directed to the corresponding author.
